# Finite Element Analysis of Foot and Ankle Impact Injury: Risk Evaluation of Calcaneus and Talus Fracture

**DOI:** 10.1371/journal.pone.0154435

**Published:** 2016-04-27

**Authors:** Duo Wai-Chi Wong, Wenxin Niu, Yan Wang, Ming Zhang

**Affiliations:** 1 Interdisciplinary Division of Biomedical Engineering, The Hong Kong Polytechnic University, Hong Kong SAR, China; 2 Shanghai Yang Zhi Rehabilitation Hospital, Tongji University School of Medicine, Shanghai, China; University of Manchester, UNITED KINGDOM

## Abstract

**Introduction:**

Foot and ankle impact injury is common in geriatric trauma and often leads to fracture of rearfoot, including calcaneus and talus. The objective of this study was to assess the influence of foot impact on the risk of calcaneus and talus fracture via finite element analysis.

**Methods:**

A three-dimensional finite element model of foot and ankle was constructed based on magnetic resonance images of a female aged 28. The foot sustained a 7-kg passive impact through a foot plate. The simulated impact velocities were from 2.0 to 7.0 m/s with 1.0 m/s interval.

**Results:**

At 5.0 m/s impact velocity, the maximum von Mises stress of the trabecular calcaneus and talus were 3.21MPa and 2.41MPa respectively, while that of the Tresca stress were 3.46MPa and 2.55MPa. About 94% and 84% of the trabecular calcaneus and talus exceeded the shear yielding stress, while 21.7% and 18.3% yielded the compressive stress. The peak stresses were distributed around the talocalcaneal articulation and the calcaneal tuberosity inferiorly, which corresponded to the common fracture sites.

**Conclusions:**

The prediction in this study showed that axial compressive impact at 5.0 m/s could produce considerable yielding of trabecular bone in both calcaneus and talus, dominantly by shear and compounded with compression that predispose the rearfoot in the risk of fracture. This study suggested the injury pattern and fracture mode of high energy trauma that provides insights in injury prevention and fracture management.

## Introduction

Foot and ankle impact injury is common in geriatric trauma. Nearly 50% of elderly patients suffered from foot and ankle injury resulting from high-energy mechanism [1] and fall is the most common mechanism to this injury [2]. Majority of this high energy impact injury would induce ankle and calcaneus fracture. Nearly one-third of foot injuries involved calcaneus fracture [1], whilst the management of calcaneus, talus and ankle fractures can be challenging. Understanding the mechanism of injury and injury pattern is crucial in the decision-making process for surgical interventions [1].

Talus and calcaneus play an important and integral role in weight-bearing; provide anchorage for other critical ligamentous structures; and integrate the paramount subtalar, ankle and tarsal joints [3]. The economic cost of rearfoot fracture is enormous. Patients were totally disabled for up to 3 years and partially impaired up to 5 years [4]. The treatments reported poor functional outcome due to the anatomical complexity, lack of vascularization, weight-bearing and continuous motion [5, 6].

Since the ankle and rearfoot are located along the load line of the lower extremity, axial compressive load predominantly contributes to the mechanism of rearfoot fracture [6, 7]. Therefore, it is necessary to understand the mechanism of rearfoot from the biomechanical point of view. Numerous studies investigated the tolerance of foot and ankle towards collision impact and experimented by high axial compressive load [7]. Cadaveric specimens were loaded to fracture with a prescribed impact velocity or energy [8]. Some studies simulated the foot on pedal during impact by loading a dorsiflexed foot [9]. Different impact forces [10], velocities [11] and foot postures [12] were also evaluated. Calcaneus fracture was the most common injury demonstrated in cadaveric studies, followed by talus and ankle fracture [6]. However, cadaveric studies reported high variations in outcome and protocols. The magnitudes of load-to-fracture in a single study could range from 3.7 to 8.3kN [6]. Different loading profile was also considered as a quasi-static scenario [12]. An impact velocity as high as 12m/s was also envisaged, mimicking a landmine explosion below the vehicle [11].

Computational models can further evaluate the influence of impact on rearfoot fracture when experiments are difficult [13]. Finite element (FE) analysis provide a versatile platform to assess the internal features of the complex foot structure in a controlled environment and study the sensitivity of different parameters that support both design and clinical applications [14–17]. Regarding to dynamic analysis, FE analysis was carried out to study car crash [13, 18], running [19, 20] and landing impact [21, 22]. In particular, Shin et al. [18] developed a foot-shank model to study the influence of forefoot impact via pedal on ligaments.

The objective of this study was to investigate the influence of impact velocity on the stress of calcaneus and talus, and thus the risk of fracture using FE analysis. An anatomically detailed FE model of foot and ankle complex was constructed and validated experimentally to carry out the dynamic analysis. A quantitative analysis would be conducted to evaluate the reaction forces and amount of bone yield at different impact velocity.

## Methods

The research was approved by The Human Subject Ethics Sub-committee of The Hong Kong Polytechnic University. The reference number is HSEARS20091216002. An informed consent statement was signed after receiving oral and written description of the experiment prior to the start of experiment.

### Model construction

The geometry of the model was reconstructed by coronal magnetic resonance images from the right foot of a healthy female (age 28; height 165 cm; body mass 54kg). A 3.0-T scanner (TrioTim, Siemens Medical Solutions, Erlangen, Germany) was used to scan the foot at neutral and non-weight-bearing conditions with an ankle-foot orthosis. The subject did not have known musculoskeletal disorder/pain and previous foot surgery. An informed consent statement was signed after receiving oral and written description of the experiment prior to the start of experiment.

**Fig 1:**
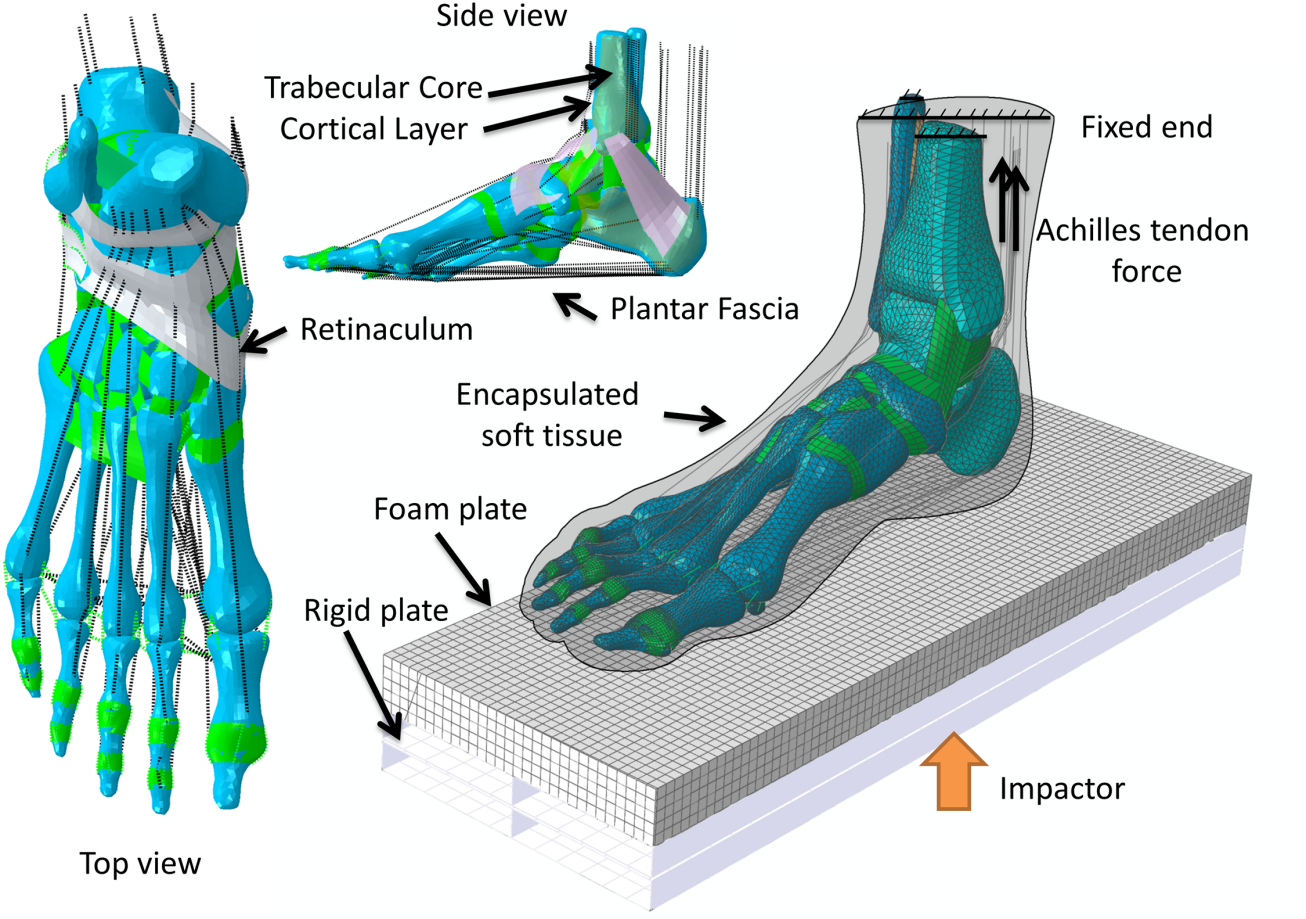
Finite element model of the foot and ankle complex. Finite element model of the foot and ankle complex showing the top and side view of the parts geometry and demonstrating the boundary and load conditions used in the simulation.

The images were segmented in Mimics v10 (Materialise, Leuven, Belgium). The segmented masks were then optimized and constructed as solid parts using Rapidform XOR2 (INUS technology Ltd., Seoul, Korea). Thirty bones and encapsulated soft tissue were finally reconstructed. Since the ankle joint is the focus of this study, the bones at the ankle were further detailed into trabecular and cortical core by a layer of 2.68mm [23]. The bones were connected to each other by contact behaviour, which was assumed frictionless [24]. Moreover, the cartilaginous layers were resembled by non-linear contact stiffness between the bones [25]. The interior of the encapsulated soft tissue was tied to the bones. Based on the constructed geometry, muscles and other soft tissues, such as ligaments and fascia, were then built through connecting the insertion points with truss, surfaces or connectors. This process was based on an anatomy atlas [26] and the model was confirmed with colleagues with expertise in anatomy. The intact model was shown as Fig 1. The modelling process also was previously described in our work on a quasi-static study [24], which also presented validation with plantar pressure study on the same participant [24], mesh convergence test [27] and cadaveric study examining the talonavicular joint force [27].

### Material properties

The material properties of the model parts were all selected from previous literatures. The elastic moduli of the trabecular and cortical were 0.4MPa and 17GPa respectively, while the Poisson’s ratios were 0.3 [28]. The elastic modulus of the bone without segmentation of trabecular and cortical was assigned with 7.3GPa and the Poisson’s ratio was 0.3 [29]. The encapsulated soft tissue [30] and skin [31] were both modelled as hyperelastic material with the second-order polynomial strain energy potential (C_10_ = 0.08556Nmm^-2^, C_01_ = -0.05841Nmm^-2^, C_20_ = 0.03900Nmm^-2^, C_11_ = -0.02319Nmm^-2^, C_02_ = 0.00851Nmm^-2^, D_1_ = 3.65273mm^2^N^-1^) and the first-order Ogden model (μ = 0.122kPa, α = 18) respectively. The elastic moduli of all ligaments were assumed 264.8MPa, which was the reported average elasticity of rearfoot ligaments [32]. The forefoot ligaments that modelled with truss were assigned with a cross-section area of 18.4mm^2^[33]. The other ligaments that modelled with surface were assigned with a thickness of 1.5mm [24, 34]. A foam padding (19-mm thick) was served as the footplate with elastic modulus of 15MPa [6], which was then attached to a rigid plate.

### Boundary and loading Conditions

The finite element simulation was carried out in Abaqus 6.11 (Dassault Systèmes, RI, USA). For the contact between the encapsulated soft tissue and ground, the coefficient of friction was set to 0.6 [35]. Two load cases were preceded for validation purpose. Firstly, the boundary and loading conditions simulated the axial compression test of a cadaveric study [6]. The proximal tibia and fibula, and the encapsulated soft tissue were fixed in all six degrees of freedom. An impactor would strike on the foot through a foot plate at 5.0 m/s (Fig 1). Since the mass of the impactor was not specified in the cadaveric study [6], it was approximated to 7 kg based on other similar studies [8, 9]. The foot plate weighed 4.5 kg, comprised of a foam part and a rigid part. A compression force of 270N (half of body weight) would be applied once the foot plate hit and came into contact with the foot.

Another load case that included the Achilles tendon force would also be resembled. The Achilles tendon would be loaded 1.94kN and the same amount of force was also added to the compressive load [6]. A parametric analysis would then be carried out with the basic setting of the same cadaveric study [6], but simulated with different impact velocities (2.0 to 7.0 m/s).

### Data analysis

With respect to the validation, the ground reaction force (GRF) and tibial reaction force (TRF) would be evaluated with and without the Achilles tendon force. TRF is the predicted reaction force at the tibia end to keep it fixed. Parametric study would be conducted on the impact velocity from 2.0m/s to 7.0m/s at 1.0m/s interval. The association of impact velocity on GRF, TRF, von Mises stress and Tresca stress of the trabecular bone in calcaneus and talus would be investigated. The volume of bone that exceeded the proposed yielding threshold would be presented to identify the risk of bone fracture. The compressive yielding stress of trabecular calcaneus was 1.8MPa [36], while the shear yielding stress was 0.792MPa, estimated by the ratio of shear-to-compressive strength [37].

## Results

### Validation

**Fig 2:**
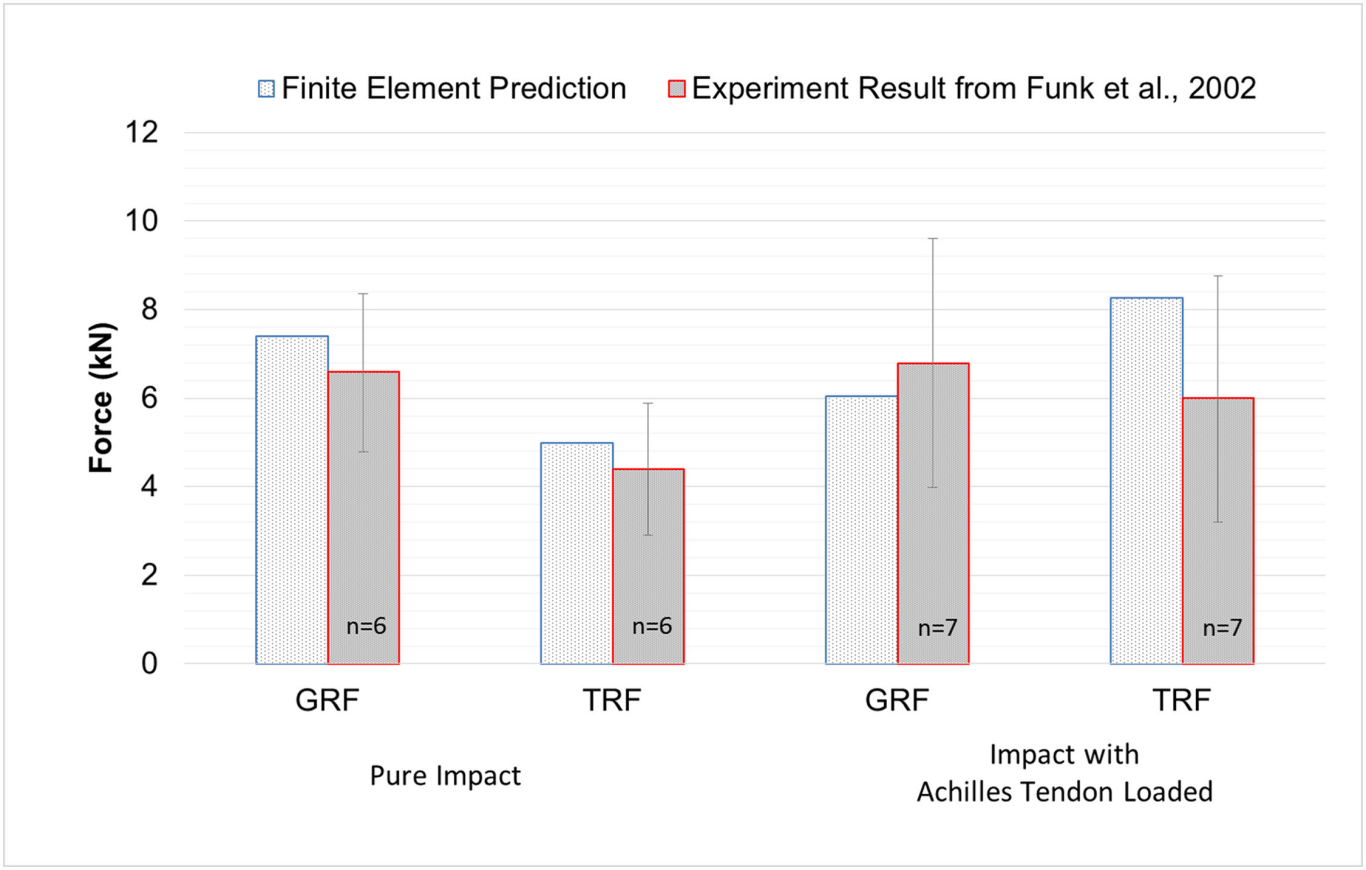
Validation of finite element model by comparing to existing literature. Comparison of finite element prediction and cadaveric experiment results from Funk et al., 2002 on ground reaction force (GRF) and tibial reaction force (TRF) under pure impact and impact with Achilles tendon loaded. (S1 Table)

As shown in Fig 2, GRF and TRF under pure impact and impact with Achilles tendon load were compared between the FE prediction and the experimental measurements of existing cadaveric study [6]. In the cadaveric study, only findings in which the specimen did not sustain a fracture were used.

The predicted GRFs were 7.4kN and 6.0kN under pure impact and Impact with Achilles tendon load. The deviations were 11.1% and 12.2% respectively. Regarding to the TRF, the difference was less than 0.6kN under the pure impact condition, while the difference was relatively large under impact with Achilles tendon loaded, accounting for 27.5%.

### Ground reaction force and tibial reaction forces

**Fig 3:**
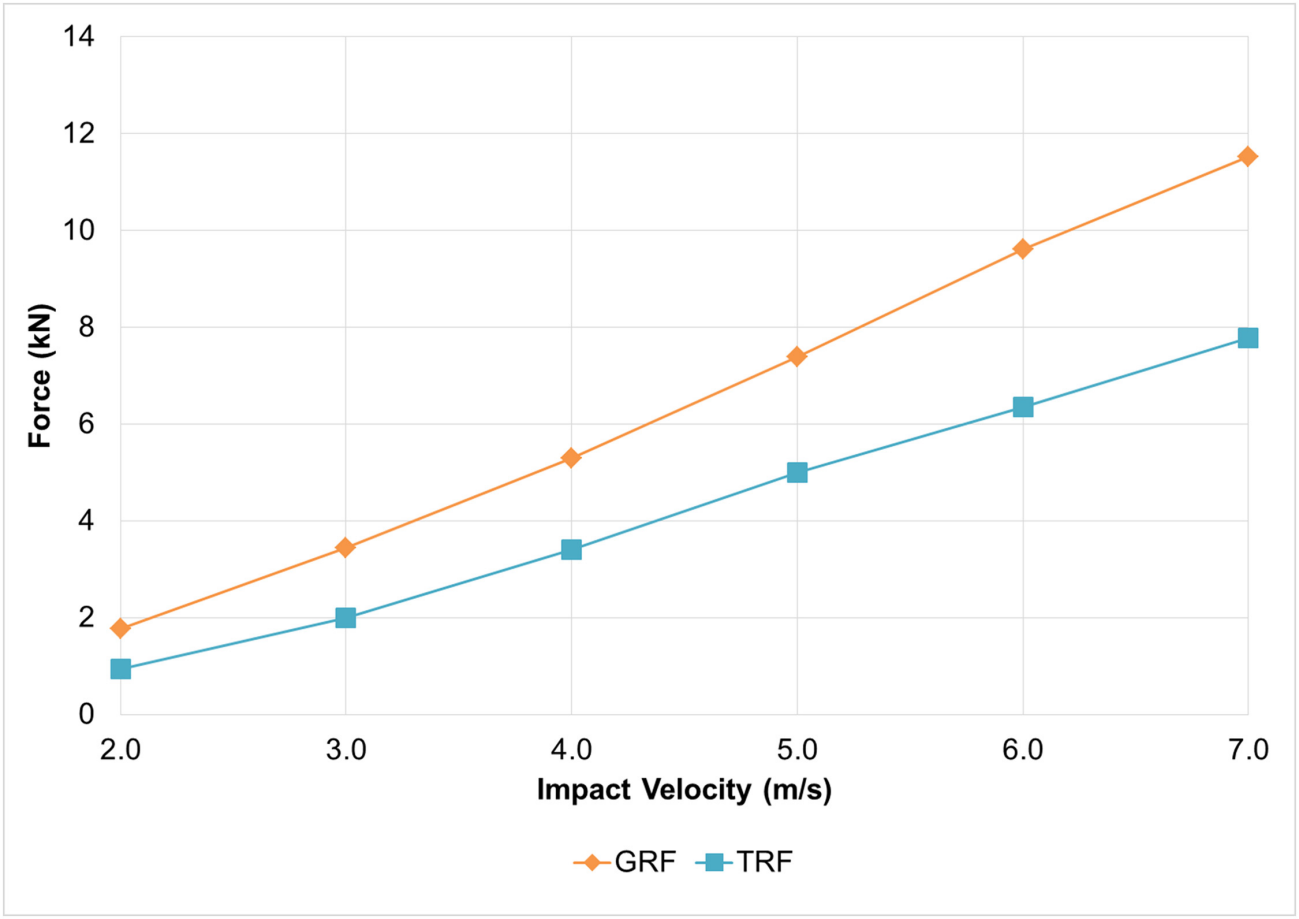
Ground reaction force (GRF) and tibial reaction force (TRF) under different impact velocity (2.0–7.0 m/s). (S2 Table).

The influence of impact velocity on GRF and TRF is presented in Fig 3. Both GRF and TRF showed a linear positive relationship with the impact, but GRF demonstrated a sharper increase compared to TRF. At 2.0m/s impact velocity, the GRF and TRF were 1.77kN and 0.94kN respectively, accounted for about 3.3 times and 1.7 times body weight. The GRF and TRG reached 11.53kN and 7.78kN at 7.0m/s impact velocity. The deviation between GRF and TRF was increased from 33% to 47% within the study range of impact velocities.

### Von Mises and Tresca stresses

**Fig 4:**
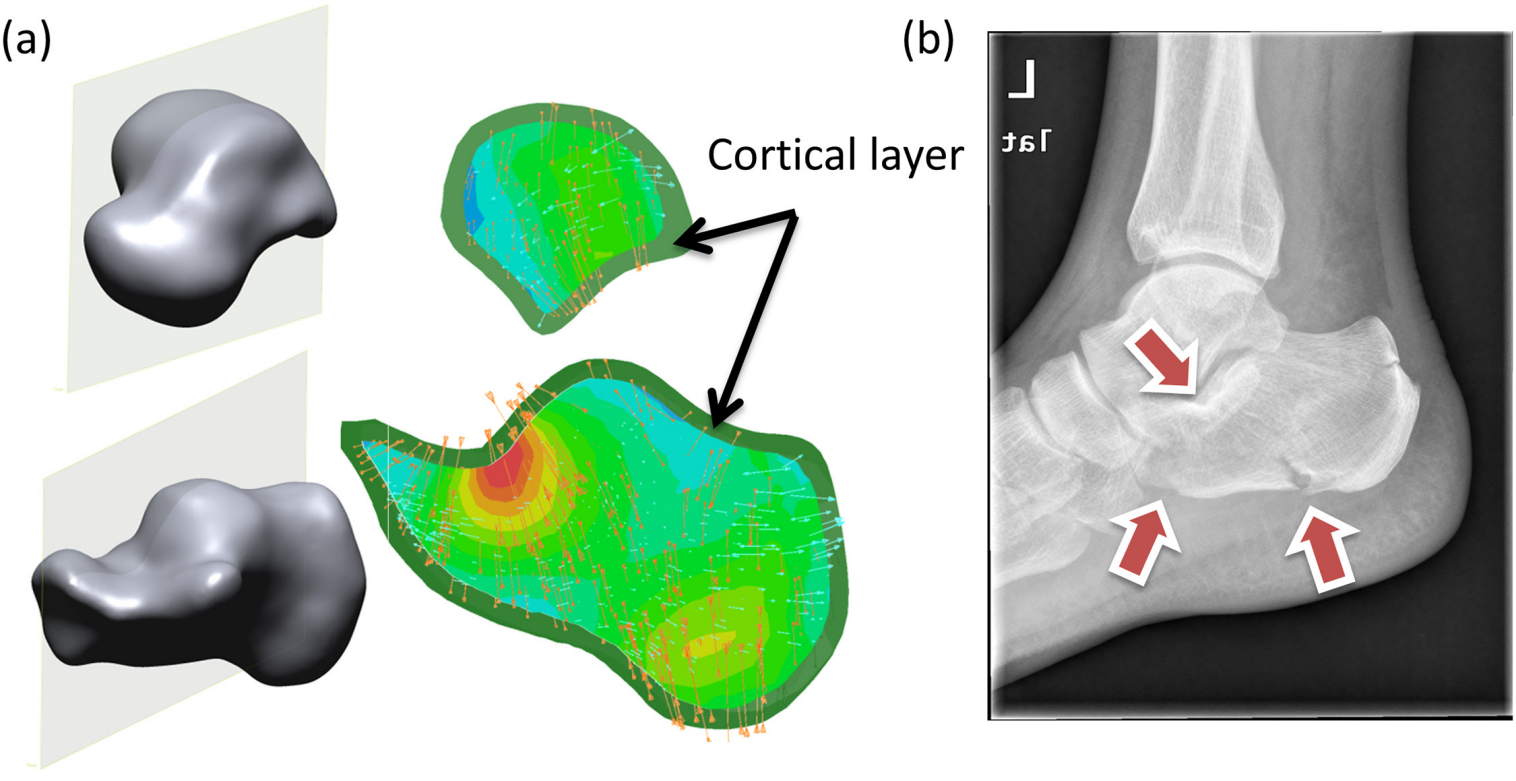
Von Mises stress of calcaneus and talus. (a) Cross section view of von Mises stress of the calcaneus and talus at 5.0 m/s impact velocity. Orange arrows indicate compressive stress. Cyan arrows indicate tensile stress. (c) X-ray of a typical patient with calcaneus fracture due to high compressive load. Arrows indicate regions of fractures.

**Fig 5:**
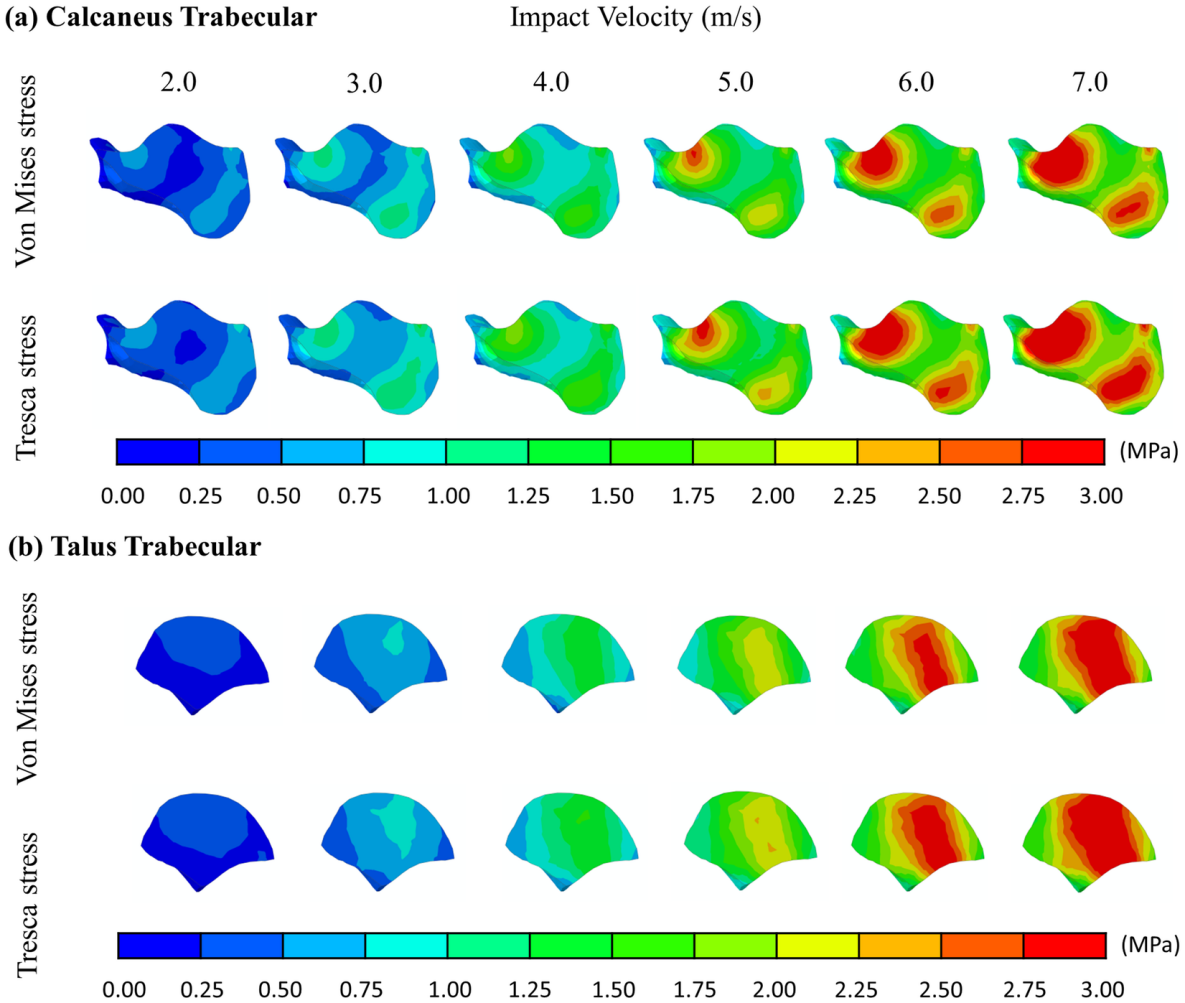
Von Mises and Tresca stresses of the calcaneus and talus trabecular at different impact velocities.

**Fig 6:**
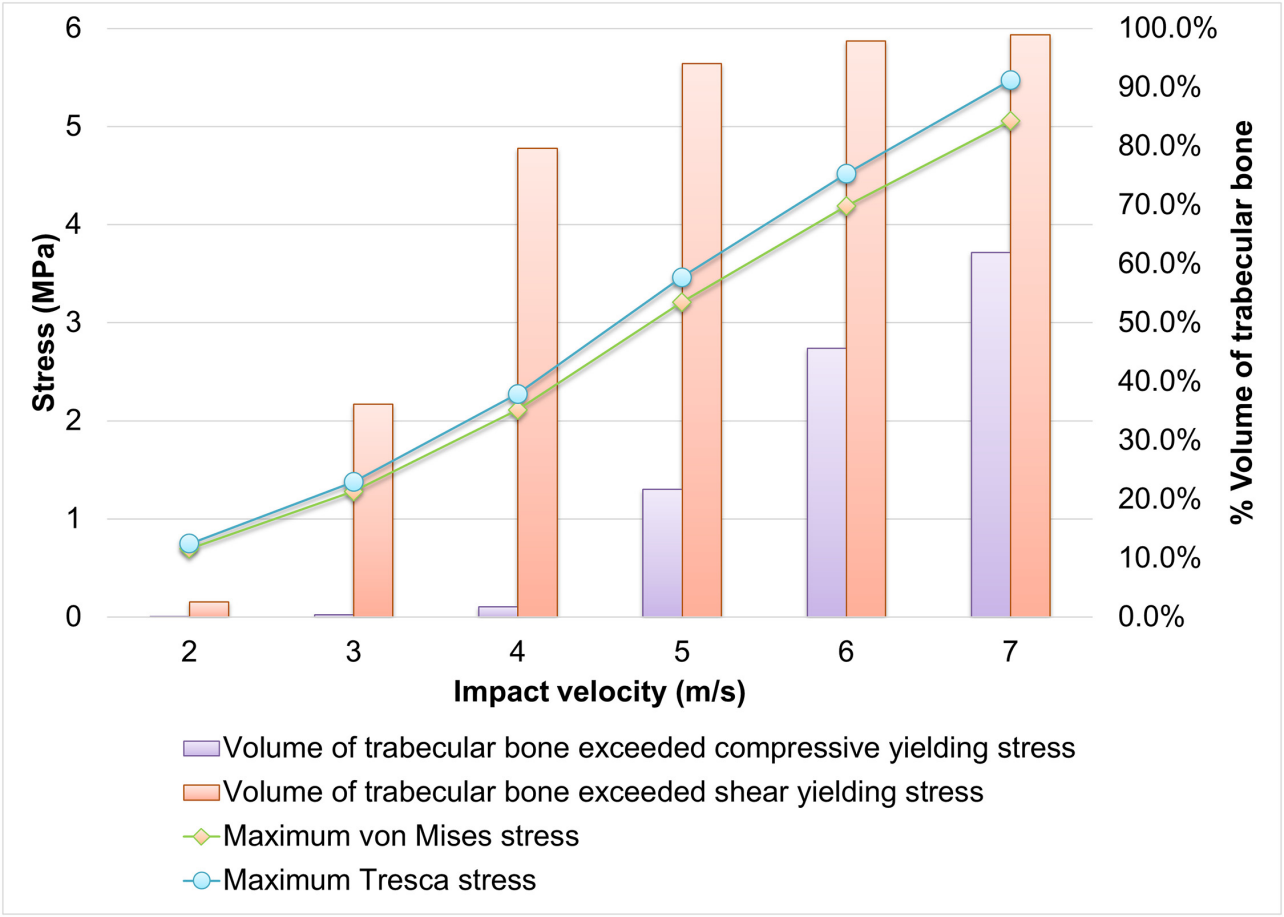
Maximum von Mises and Tresca stress with yielding volume of trabecular calcaneus against impact velocity. (S3 Table).

Fig 4a shows the von Mises stress of the calcaneus and talus. The regions of peak stresses corresponded to the fracture sites of a patient as shown in Fig 4b. Both maximum von Mises stress and Tresca stress of the trabecular calcaneus followed the increasing trend of impact velocity as shown in Figs 5 and 6. The maximum Tresca stress was always higher than the maximum von Mises stress with a minimal deviation of 7% at 2.0 m/s impact. The maximum von Mises stress increased from 0.70 MPa to 5.06 MPa when the impact velocity increased from 2.0 m/s to 7.0 m/s, while that of Tresca stress increased from 0.75MPa to 5.47MPa.

The volume of trabecular calcaneus that exceeded yield is presented in Fig 6. The trabecular calcaneus underwent shear yielding predominantly compared to compressive yield. The volume of bone with shear yielding increased considerably from 36.2% at 3.0 m/s impact to 79.7% at 4.0 m/s. Nearly all trabecular bone of the calcaneus exceeded the yielding point of shear at 7.0 m/s impact. Trabecular bone that exceeded the compressive strength was relatively mild. About one-fifth and two-third of the volume reached the compressive yield at 5.0 m/s and 7.0 m/s impact respectively.

**Fig 7:**
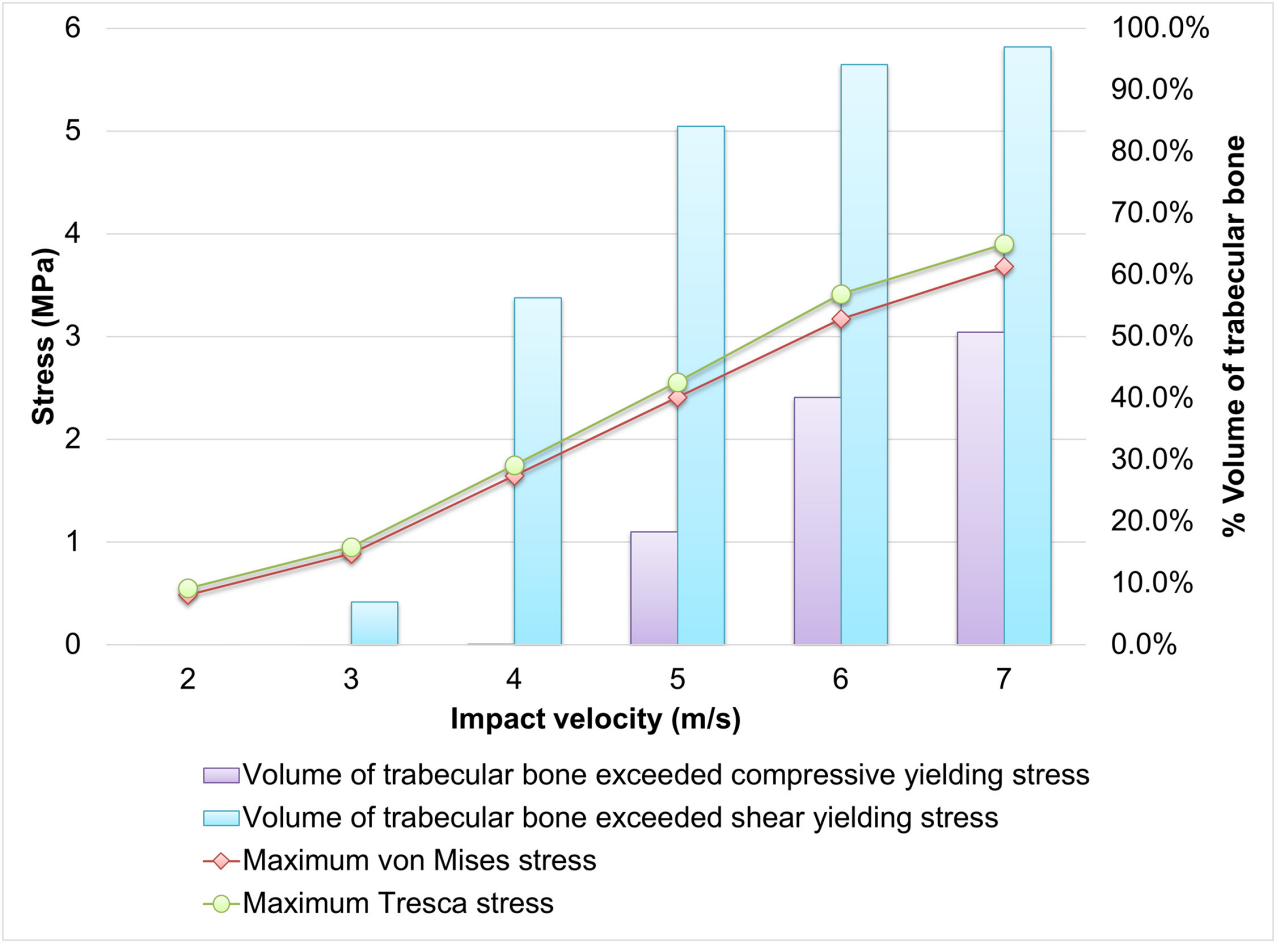
Maximum von Mises and Tresca stress with yielding volume of trabecular talus against impact velocity. (S4 Table).

The maximum von Mises stress and Tresca stress of trabecular talus are shown on Figs 5 and 7. Similar to that of calcaneus, the stresses increased with increasing impact velocity and the maximum Tresca stress was always higher than the maximum von Mises stress. The maximum von Mises stress and Tresca stresses were 0.48MPa and 0.55MPa at 2.0 m/s impact respectively. The stresses increased to 3.68MPa and 3.90MPa at 7.0m/s impact. Compressive yield apparently started at 5.0 m/s impact with about 18% total volume and increased to about half the bone at 7.0 m/s. Shear yield was minimal at 3.0 m/s while increased to about half the bone at 4.0 m/s.

## Discussion

High energy foot and ankle injuries are commonly caused by falls and remain prevalent to elderly [38]. Elderly people could fall from standing height due to declined visual-auditory functions, proprioceptive input and muscle weakness. Healthy and active older adults have even higher risk of falls since they are likely to perform more dangerous activities, such as climbing a ladder or roof [1]. In fact, high energy foot and ankle injuries also happen in other scenarios. For example, automotive intrusion is the second leading cause of high energy trauma [2] that is caused by high-speed impact of the pedal on the restrained foot. Besides, some risky sport activities and encounters such as skiing, parachuting and underbody blast contribute to this type of injury [39, 40]. Understanding the mechanism and of high energy trauma not only help in the management of fracture, but also establish assessment index and strategy for designing preventive measures [39].

Parametric analysis of different contributing factors was essential to understand the mechanism of high energy trauma of the rearfoot. Computational simulation can provide a versatile platform to investigate different factors in a controlled environment. In this study, FE model of the entire foot and ankle was developed and parametric analysis on the impact velocity was carried out. The anatomically-detailed foot and ankle constructed in this study is prevailing, since the reactions and tensions of soft tissue, ligamentous and adjacent bony structures were taken into consideration.

Besides the validation conducted in our previous work [24, 27], the simulation was validated and compared to a cadaveric experiment with similar settings in this study [6]. During pure impact, both FE prediction and cadaveric experiment showed a smaller TRF than the GRF, presenting generally agreeable findings. Other studies reported maximum measured forces ranging from about 3.8kN to 9.8kN in different experiment protocols [6, 11, 41]. On the contrary, there was a relatively larger deviation under the condition of impact with the Achilles tendon load. This could be due to the variation in the application of Achilles tendon force. The prediction in this study adopted an average value of the tendon force (1940N), while the Achilles tendon force ranged from 936N to 2644N in the experiment.

Calcaneus fracture is dominant in axial compressive impact [6] and the chance of injury increases with greater impact velocity [8], which was also advocated by the percentage yielding of the bone demonstrated in this study. More than 90% of the trabecular in calcaneus suffered shear yielding together with about 20% of compressive stress yielding at 5.0 m/s. In fact, the foot impact velocity of walking and running ranges between 0.52 m/s and 0.72m/s [42]. During risky sport activities, such as skiing, the impact velocity could reach 14.0 m/s at 10-m falling height when the landing angle is inappropriate [43]. On the other hand, the foot would sustain 3.0 m/s to 14.0 m/s pedal intrusions during a frontal car crash [44]. A number of studies attempted to establish measures and standards for landing safety, parachutes and aircraft [43, 45].

The combined shear and compressive yielding would be alarming despite that the volume of compressive yield was relatively small. The risk of injury with 5.0 m/s impact velocity was also demonstrated [6, 8, 9]. About 93% of specimen sustained calcaneus fracture with 5.0 m/s impact [6]. Gallenberger et al. [8] found calcaneus fracture in all specimens with an average impact velocity of 6.3 m/s. The 50% probability of injury demonstrated in various cadaveric studies ranged from 6100 N to 9300 N [8]. While the predicted TRF on the risk of bone fracture in this study was smaller than the proposed range, yielding stresses were used in this study instead of ultimate stress to present a more conservative injury criterion of loading.

Both von Mises and Tresca stresses of the calcaneus concentrated at the talus articulation and the inferior calcaneus tuberosity (Fig 5). The findings corresponded to the fracture sites and pattern found in cadaveric studies [6, 11]. On the other hand, talus is the second most frequent fracture site during the compressive impact [11]. The concentrated stress at the superior trochlea of talus and the posterior talocalcaneal articulation reflected the injury sustained in these locations (Figs 4 and 5), while anterior talocalcaneal articulation and lateral malleolar articulation fracture could be secondary to the fractured calcaneus and the displaced talus. The prediction also showed that calcaneus and talus fracture appeared to develop at the same impact velocity. Although the stresses of the talus were smaller than that of the calcaneus, the volumes of yielded trabecular bone in both compression and shear were similar. Besides, the constitutive model of the bone material was assumed elastic in our simulation, while it should be better characterized by elasto-plastic behaviour [46]. Evaluating the predictions with material yield tends to overestimate the risk of fracture.

Shear failure was demonstrated as the major failure mode in foot impact, while higher axial impact velocity would result in a combined compressive-shear failure. Both Figs 4 and 6 indicated that the maximum Tresca stress of the trabecular were higher than the von Mises stress. Since the shear strength of trabecular bone was weaker than the compressive strength [37], the volume of bone stock undergoing shear yield was considerably higher than that of compressive yield. However, it should be noted that the bone may be subject to more plastic deformation in shear compared to compression before reaching the ultimate strength [47].

Bone fracture criterion is an important indicator to identify the risk of failure, but there is a lack of agreement among different studies. The maximum stress criterion assumes failure appeared when the principal stress is higher than the ultimate strength, while principal strain (Saint-Venant criterion) is also recommended [48]. In this study, the distortion energy criteria with both von Mises-Hencky criterion and Tresca criterion were adopted. It was suggested that the von Mises-Hencky criterion produces the most accurate result when material properties are assumed isotropic [48]. While the von Mises-Hencky criterion is not robust on shear, the inclusion of Tresca criterion could compensate its drawback [48].

There were some limitations on the context of model simplifications and settings. The trabecular and cortical bone was assumed homogeneous and isotropic. The analysis of cortical bone was not conducted because trabecular bone fails before cortical bone under compression generally [49]. In addition, the trabecular and cortical core of the midfoot and forefoot were not segmented. The Achilles tendon and plantar fascia was modelled with trusses that may result in stress concentration at the insertions of calcaneal tuberosity. Besides, the influence of muscle force was disregarded in the parametric study. Tibia fracture was not studied, since it was secondary to calcaneus or talus fracture and was not the critical element in axial compressive impact [6].

External validity remains a challenge in finite element analysis that hinders the generalization of the findings. Since creating a single model and simulation could be strenuous, most of the finite element studies adopted a single-subject design [15, 28]. This study endeavoured to invite a healthy young female that believed to be typical and representative. On the other hand, some of the constitutive models of materials were simplified. For example, the hypelastic behaviour of foam was simplified as elastic. The viscous behaviour of soft tissue was also neglected that may impose some inaccuracies to the predictions.

Most cadaveric studies adopted a survivorship analysis to establish a 50% probability of injury criterion. The mass of impactor could vary from 3.3 kg to16 kg and produce different injury and sub-injury responses [8, 9]. In computer simulations, a factor analysis can be conducted to observe the power of importance in different determinants, such as impact velocity and impact energy. Future work could also investigate the influence of muscle force, foot posture, foot support and impact mass on the risk of rearfoot fracture.

In conclusion, the prediction in this study showed that axial compressive impact at 5.0 m/s could produce considerable yielding of trabecular bone in both calcaneus and talus, dominantly by shear and compounded by compression that predispose the rearfoot in the risk of fracture. In the future, this computational platform can investigate the relationship between different loading modes and fracture pattern/mechanism that could support design for the prevention of injury, as well as management of the fracture.

## Supporting Information

S1 Table Supplementary Data for Fig 2. Comparison of finite element prediction and cadaveric experiment results from Funk et al., 2002 on ground reaction force (GRF) and tibial reaction force (TRF) under pure impact and impact with Achilles tendon loaded.
(DOCX)

S2 Table Supplementary Data for Fig 3. Ground reaction force (GRF) and tibial reaction force (TRF) under different impact velocity (2.0–7.0 m/s).
(DOCX)

S3 Table Supplementary Data for Fig 6. Maximum von Mises and Tresca stress with yielding volume of trabecular calcaneus against impact velocity.
(DOCX)

S4 Table Supplementary Data for Fig 7. Maximum von Mises and Tresca stress with yielding volume of trabecular talus against impact velocity.
(DOCX)
