# Supplementary material for: Finite Element Analysis of Foot and Ankle Impact Injury: Risk Evaluation of Calcaneus and Talus Fracture
Source: PLoS One. 2016 Apr 27;11(4):e0154435. doi: 10.1371/journal.pone.0154435 (PMC4847902; doi:10.1371/journal.pone.0154435)
Supplement: S1 Table — Comparison of finite element prediction and cadaveric experiment results from Funk et al., 2002 on ground reaction force (GRF) and tibial reaction force (TRF) under pure impact and impact with Achilles tendon loaded. (DOCX) [file pone.0154435.s001.docx]

**S1 Table.** **Supplementary Data for Figure 2.**

Validation of finite element model by comparing to existing literature. Comparison of finite element prediction and cadaveric experiment results from Funk et al., 2002 on ground reaction force (GRF) and tibial reaction force (TRF) under pure impact and impact with Achilles tendon loaded.

|  |  | Finite Element Prediction in this study | Experiment Results from Funk et al., 2002 | Standard Deviation of Experiment Results |
| --- | --- | --- | --- | --- |
| Pure Impact | GRF | 7.39621 | 6.578167 | 1.780251 |
|  | TRF | 4.98729 | 4.3915 | 1.486623 |
| Impact with Achilles Tendon Loaded | GRF | 6.04746 | 6.786857 | 2.81227 |
|  | TRF | 8.25994 | 5.987429 | 2.779569 |

GRF: Ground Reaction Force; TRF: Tibial Reaction Force.

Unit: kN
