# Supplementary material for: Finite Element Analysis of Foot and Ankle Impact Injury: Risk Evaluation of Calcaneus and Talus Fracture
Source: PLoS One. 2016 Apr 27;11(4):e0154435. doi: 10.1371/journal.pone.0154435 (PMC4847902; doi:10.1371/journal.pone.0154435)
Supplement: S2 Table — Ground reaction force (GRF) and tibial reaction force (TRF) under different impact velocity (2.0–7.0 m/s). (DOCX) [file pone.0154435.s002.docx]

**S2 Table.** **Supplementary Data for Figure 3.**

Ground reaction force (GRF) and tibial reaction force (TRF) under different impact velocity (2.0 - 7.0 m/s).

| Impact Velocity (m/s) | GRF (kN) | TRF (kN) |
| --- | --- | --- |
| 2.0 | 1.77407 | 0.94022 |
| 3.0 | 3.43638 | 1.99197 |
| 4.0 | 5.29516 | 3.39753 |
| 5.0 | 7.39621 | 4.98729 |
| 6.0 | 9.61551 | 6.35153 |
| 7.0 | 11.5277 | 7.77866 |

GRF: Ground Reaction Force; TRF: Tibial Reaction Force.
