# Supplementary material for: Finite Element Analysis of Foot and Ankle Impact Injury: Risk Evaluation of Calcaneus and Talus Fracture
Source: PLoS One. 2016 Apr 27;11(4):e0154435. doi: 10.1371/journal.pone.0154435 (PMC4847902; doi:10.1371/journal.pone.0154435)
Supplement: S3 Table — Maximum von Mises and Tresca stress with yielding volume of trabecular calcaneus against impact velocity. (DOCX) [file pone.0154435.s003.docx]

**S3 Table.** **Supplementary Data for Figure 6**

Maximum von Mises and Tresca stress with yielding volume of trabecular calcaneus against impact velocity.

| Impact Velocity (m/s) | Maximum von Mises stress (MPa) | Maximum Tresca Stress (MPa) | % Volume of trabecular bone exceeded compressive yielding stress | % Volume of trabecular bone exceeded shear yielding stress |
| --- | --- | --- | --- | --- |
| 2 | 0.696 | 0.749 | 0.02% | 2.58% |
| 3 | 1.279 | 1.379 | 0.42% | 36.22% |
| 4 | 2.112 | 2.276 | 1.76% | 79.68% |
| 5 | 3.208 | 3.463 | 21.73% | 94.02% |
| 6 | 4.191 | 4.52 | 45.70% | 97.89% |
| 7 | 5.06 | 5.474 | 61.95% | 98.96% |
