# Supplementary material for: Finite Element Analysis of Foot and Ankle Impact Injury: Risk Evaluation of Calcaneus and Talus Fracture
Source: PLoS One. 2016 Apr 27;11(4):e0154435. doi: 10.1371/journal.pone.0154435 (PMC4847902; doi:10.1371/journal.pone.0154435)
Supplement: S4 Table — Maximum von Mises and Tresca stress with yielding volume of trabecular talus against impact velocity. (DOCX) [file pone.0154435.s004.docx]

**S4 Table.** **Supplementary Data for Figure 7.**

Maximum von Mises and Tresca stress with yielding volume of trabecular talus against impact velocity.

| Impact Velocity (m/s) | Maximum von Mises stress (MPa) | Maximum Tresca Stress (MPa) | % Volume of trabecular bone exceeded compressive yielding stress | % Volume of trabecular bone exceeded shear yielding stress |
| --- | --- | --- | --- | --- |
| 2 | 0.484 | 0.549 | 0.00% | 0.00% |
| 3 | 0.884 | 0.949 | 0.00% | 6.95% |
| 4 | 1.643 | 1.747 | 0.10% | 56.30% |
| 5 | 2.406 | 2.554 | 18.34% | 84.11% |
| 6 | 3.17 | 3.412 | 40.09% | 94.11% |
| 7 | 3.682 | 3.901 | 50.68% | 96.99% |
